# MHC-Dependent Mate Selection within 872 Spousal Pairs of European Ancestry from the Health and Retirement Study

**DOI:** 10.3390/genes9010053

**Published:** 2018-01-22

**Authors:** Zhen Qiao, Joseph E. Powell, David M. Evans

**Affiliations:** 1 University of Queensland Diamantina Institute, Translational Research Institute, Brisbane, Queensland 4102, Australia; z.qiao1@uq.edu.au; 2 Institute for Molecular Biosciences, University of Queensland, Brisbane, Queensland 4072, Australia; j.powell@imb.uq.edu.au; 3 Medical Research Council (MRC) Integrative Epidemiology Unit, School of Social & Community Medicine, University of Bristol, Bristol BS8 1TH, UK

**Keywords:** disassortative mating, non-random mating, major histocompatibility complex, human leukocyte antigen, mate selection

## Abstract

Disassortative mating refers to the phenomenon in which individuals with dissimilar genotypes and/or phenotypes mate with one another more frequently than would be expected by chance. Although the existence of disassortative mating is well established in plant and animal species, the only documented example of negative assortment in humans involves dissimilarity at the major histocompatibility complex (MHC) locus. Previous studies investigating mating patterns at the MHC have been hampered by limited sample size and contradictory findings. Inspired by the sparse and conflicting evidence, we investigated the role that the MHC region played in human mate selection using genome-wide association data from 872 European American spouses from the Health and Retirement Study (HRS). First, we treated the MHC region as a whole, and investigated genomic similarity between spouses using three levels of genomic variation: single-nucleotide polymorphisms (SNPs), classical human leukocyte antigen (HLA) alleles (both four-digit and two-digit classifications), and amino acid polymorphisms. The extent of MHC dissimilarity between spouses was assessed using a permutation approach. Second, we investigated fine scale mating patterns by testing for deviations from random mating at individual SNPs, HLA genes, and amino acids in HLA molecules. Third, we assessed how extreme the spousal relatedness at the MHC region was compared to the rest of the genome, to distinguish the MHC-specific effects from genome-wide effects. We show that neither the MHC region, nor any single SNPs, classic HLA alleles, or amino acid polymorphisms within the MHC region, were significantly dissimilar between spouses relative to non-spouse pairs. However, dissimilarity in the MHC region was extreme relative to the rest of genome for both spousal and non-spouse pairs. Despite the long-standing controversy, our analyses did not support a significant role of MHC dissimilarity in human mate choice.

## 1. Introduction

Assortative mating occurs when individuals tend to reproduce with mates who are either similar (known as positive assortment or homogamy) or dissimilar (known as negative assortment or disassortative mating) to themselves. In human populations, large-scale studies have shown that the overwhelming majority of assortment is positive [1,2,3,4]. Spousal correlations indicate high positive assortment on traits such as religion and social attitudes, moderate assortment on intelligence, and low assortment for many physical traits including height [5]. The only well-documented example of negative assortment in humans is reported to involve spousal dissimilarity at human leukocyte antigen (HLA) alleles within the major histocompatibility complex (MHC) region, although this is controversial and the evidence has been conflicting [6,7,8,9,10].

The MHC is a highly polymorphic region of the human genome located on the short arm of chromosome six that extends approximately 4.97 Mb. The MHC contains many genes that encode cell surface molecules involved in immune recognition and response to specific pathogens, including the human leukocyte antigen (HLA) genes that code for cell surface receptors that present foreign and self-peptides to the adaptive arm of the immune system [6,11]. The high polymorphism and great diversity within the MHC in humans and other species has been hypothesized to be a result of several evolutionary forces including balancing selection and potentially sexual selection and disassortative mating [7,12].

Negative assortment at MHC loci has been observed in a number of vertebrates [13], such as fish [14,15], reptiles [16], birds [17,18], rodents [19,20,21], and other species [22]. One hypothesis is that since negative assortment increases heterozygosity at HLA loci, this greater diversity will improve offspring’s ability to respond to a greater range of pathogens, and consequently confer a survival advantage. Although the existence of disassortative mating is well-established in animals, evidence for MHC-based mate choice in humans is highly controversial [6,8,9] and was based at least initially upon indirect evidence obtained from so called “sweaty T-shirt” studies that showed women were found to prefer T-shirts worn by MHC-dissimilar men [23]. 

More recently, genomic studies have been conducted to directly measure HLA similarity among real-world couples. The results of these studies have been conflicting. Many have found no strong evidence of disassortative effects of the MHC on mate choice [24,25,26,27,28,29], whilst others have shown dissimilarity [30] or even excess MHC similarity among spouses [31]. The availability of genome-wide data has also allowed researchers to investigate MHC-dependent mate choice at better resolution, as well as to contrast genetic similarity between spouses across the MHC region from similarity across the rest of the genome. In 2008, Chaix et al. found that 28 European American couples in the HapMap 2 dataset were significantly more MHC dissimilar than random pairings of individuals in the same dataset, suggesting an important role of the MHC in mate selection (although the same authors found no evidence of excess MHC dissimilarity between African spouses) [6]. Shortly after, Derti et al. failed to replicate this analysis in the HapMap phase 2 dataset, or to validate the results in the larger HapMap phase 3 sample [8]. However, both of these studies utilized small samples, which may not be large enough to demonstrate negative assortment reliably and are based on a cultural isolate (i.e., Mormons in Utah), which may not be generalizable to other populations. In contrast, in a larger sample of 930 couples with children afflicted with multiple sclerosis (MS), Khankhanian et al. reported excess dissimilarity in the MHC II region, but excess similarity in the MHC I region [32]. However, this dataset may not be representative of the general population and may be subject to spurious correlations resulting from the ascertainment on affected offspring with multiple sclerosis. A very recent review of MHC-dependent mate selection in human and non-human primates found some evidence for preferential selection of MHC diverse mates (i.e., individuals with high MHC heterozygosity were most desirable) but no overall effect of MHC dissimilarity on mating decisions in humans [33].

Driven by these apparently contradictory findings, we investigated MHC-dependent mate choice in a large, independent sample of individuals from the Health and Retirement Study [34]. In addition to investigating overall genomic similarity across the entire MHC region, we also tested couples for increased/decreased similarity at individual single-nucleotide polymorphisms (SNPs), classical HLA alleles, and HLA amino acids. Our rationale for examining fine scale patterns of variation was that different regions of the MHC may exhibit varying levels of similarity/differentiation and that examining all variants in the MHC region in combination may not accurately represent the biological significance of individual loci.

## 2. Materials and Methods

### 2.1. Data

Publicly available data from the Health and Retirement Study (HRS; dbGaP Study Accession phs000428.v1.p1) was used to investigate genetic similarity between spouse pairs. The HRS consists of 12,507 individuals genotyped on the Illumina HumanOmni2.5-4v1 array (Illumina, San Diego, CA, USA) [34]. According to the documentation supplied with the dataset, 8652 individuals were of European ancestry, and this was confirmed by principal components analysis applied to genome-wide SNP data (Figure S1). We removed 1000 of the 8652 individuals with estimated genome-wide SNP relatedness greater than 0.025 (roughly equal to 5th degree relatives) as calculated by the GCTA software [35,36,37]. Of the remaining individuals, there were 872 opposite-sex spousal pairs (where cohabitation was treated the same as marriage), which form the basis of all analyses presented here. All couples were born between 1910 and 1960, and more than 90% were born in the 1930s and 1940s. Strict quality control (QC) procedures were performed to ensure only common, high quality genotypes were used in the analysis: no SNPs failed Hardy–Weinberg equilibrium test (HWE *p*-value < 10^−6^) and 75,717 SNPs were removed due to a missing rate greater than 0.01. After HLA-imputation (see below), a further 937,969 SNPs were removed from analyses because they had a minor allele frequency (MAF) less than 0.05. After individual and marker QC, 1,138,428 autosomal SNPs remained. In this dataset, 4678 SNPs were located in the MHC region (defined as positions 28,477,797–33,448,354 on chromosome 6, in GRCh37/hg19 genome builds) [38].

The classical HLA alleles, and HLA amino acid polymorphisms in HLA genes, were imputed based on the SNPs in the MHC region using the software package SNP2HLA [39]. Eight HLA genes (three class I genes: *HLA-A*, *-B*, *-C*, five class II genes: *HLA-DPA1*, *-DPB1*, *-DQA1*, *-DQB1*, and *-DRB1*) were imputed. The HLA alleles were imputed for both two- and four-digit classifications, and the identity of 399 amino acid sequences/residues located within the HLA region was inferred using the four-digit HLA allele information. We then chose HLA types for genes and amino acids for each individual based on the imputed posterior probabilities. If the imputed probabilities for an individual implicated more than two possible alleles/amino acids (i.e., multiallelic sites), then the genotype was set to missing. The missing rate for individual HLA genes and amino acids refers to the percentage of individuals having at least one missing allele at this locus (Tables S1–S3). Twenty three out of 399 amino acid residues with a missing rate greater than 1% were removed from the downstream analyses. SNP2HLA has the advantage of enabling researchers to potentially identify functional coding variants that might be causal for complex diseases [39].

### 2.2. Statistical Analysis

#### 2.2.1. Major Histocompatibility Complex Region as a Whole

We first treated the MHC region as a whole, and investigated mate selection patterns using three levels of genomic variation separately (i.e., three different analyses): SNPs, classical HLA alleles, and amino acid polymorphisms. We then tested for any deviations from random mating at individual SNPs, HLA genes, and HLA amino acids in order to investigate whether there was any evidence for fine scale mate selection patterns.

Following previous studies [6,8], the proportion of identical variants between a pair of individuals was quantified using the identity coefficient (*Q*). For a given SNP, *Q* was 0 if both individuals were homozygous and carrying a different allele (e.g., AA and GG); *Q* was 1 if both individuals were homozygous and carrying the same allele (e.g., AA and AA) or heterozygous and carrying the same alleles (e.g., AG and AG); and *Q* was 0.5 in all other cases. In the case of a given classical HLA gene or amino acid residue, we defined the identity coefficient as 0 if the two individuals shared 0 HLA alleles or amino acid residues in common, 0.5 if they shared only one allele or amino acid residue in common, and 1 in all other cases.

The relatedness coefficient *R* was defined as *R_a,b_ = (Q_a,b_ − Q_m_)/(1 − Q_m_)* where *Q_a,b_* is the identity coefficient for a given pair of individuals (*a*, *b*), and *Q_m_* is the identity coefficient averaged over all possible opposite-sex pairs of individuals in the study sample (we note that this differs slightly from the definition of *Q_m_* as defined in Chaix et al. [6] who calculate *Q*_m_ over all individuals, not just opposite sex pairs). Positive values of *R_a,b_* indicate genetic similarity between individuals *a* and *b*, whereas negative values imply genetic dissimilarity between them relative to random opposite-sex pairs of individuals in the sample. The mean (*R_mean(spouse)_*) and median (*R_median(spouse)_*) values of *R* were derived for all spousal pairs in the HRS. 

The genetic relatedness coefficient *R* was calculated across the MHC region for each of three levels of genomic variation: SNPs, classical HLA genes (both four-digit and two-digit classifications), and amino acids. Namely, identity coefficients for each couple (*Q_a,b_*) were first calculated across 4678 SNPs, 8 HLA allelic types, or 376 HLA amino acids residues, and the corresponding *Q_m_* was obtained by averaging over all possible opposite-sex pairings. Then, the relatedness coefficient between couples (*R_a,b_*) at different levels could be computed using the corresponding *Q_a,b_* and *Q_m_*. Note that *R* was a multilocus relatedness coefficient when using classical HLA alleles or amino acid data.

The extent of MHC dissimilarity between spouses was assessed using a permutation approach. In each permutation, females were randomly paired with males and relatedness coefficients were calculated for each pairing. The mean and median relatedness coefficients (*R_mean(permutations)_* or *R_median(permutations)_*) were then calculated across all couples for each permutation and a two-sided *p*-value was calculated. The two-sided *p*-value (*p*) was equal to the proportion of permutations in which the mean/median of the permutated couples was the same or more extreme than the mean/median relatedness across the actual couples; 100,000 permutations were performed. Real couples were allowed in permutations.

#### 2.2.2. Genomic Similarity across the MHC Compared to Genomic Similarity across the Genome

The permutation approach described above was used to assess whether MHC relatedness among couples was more dissimilar than MHC relatedness amongst random pairings of opposite sex individuals. However, we were also interested in how extreme spousal relatedness at the MHC region was compared to the rest of the genome.

Following previous studies [6,8], we calculated the recombination rates and relatedness coefficients between spousal pairs for sliding windows of 4.97 Mb (i.e., the same size as the MHC region) across the genome. Recombination rates were obtained from phase 2 of the International HapMap Project, which were lifted over to Hg19 [40], and the centromere base positions were obtained from the UCSC Genome Browser web site (http://genome.ucsc.edu). The detailed steps for this analysis were as follows: first, the genome (except chromosomes 6, X and Y) was divided into segments of 4.97 Mb in overlapping increments of 300 Kb. Segments with less than 1000 SNPs or those overlapping a centromere were excluded. The rationale for removing chromosome 6 was to ensure that the ‘background’ genomic windows included neither the MHC region nor a region in linkage disequilibrium (LD) with the MHC. Second, the recombination rate (cM/Mb) was calculated by dividing the difference in genetic distances (which reflects recombination frequency) between the two positions at each end of the genomic window by 4.97 Mb. To account for the low linkage disequilibrium across the MHC, we only utilized segments with recombination rates the same or lower than the MHC region. We randomly sampled 10,000 genomic windows from the remaining windows. We then compared the mean and median relatedness coefficients between spousal pairs across the MHC region against similar-sized windows across the genome that met the above criteria. We also examined genomic similarity in the case of opposite-sex non-spousal pairs.

#### 2.2.3. Individual SNPs, HLA Alleles, and Amino Acids

We further investigated MHC dissimilarity at finer scales. First, in order to detect evidence of significantly dissimilar SNPs within the MHC region, the Spearman’s rank correlation coefficient *ρ* was used to measure relatedness between spousal pairs based on their SNP genotypes (coded as 0, 1, and 2) at individual SNPs. For HLA genes and amino acid residues, relatedness was measured using a similarity score *SC*, which was calculated by counting how many copies of HLA alleles or amino acids were shared between each spouse pair (i.e., 0, 1, or 2) and then summed across all spouses [32]. Thus, a *SC* could be obtained for each of the 8 classical HLA alleles and 376 amino acid residues. Significance was assessed using a normal distribution, with mean and standard deviation estimated from 100,000 random permutations (where the spouses were reordered); all *p*-values were two-sided.

## 3. Results

In this study we investigated genetic signatures of non-random mating patterns using several complementary approaches. First, we used the relatedness coefficient *R* to investigate genetic similarity between spouses compared to random combinations of opposite sex individuals across the MHC region. We also compared the degree of similarity between spouses in the MHC region to the rest of the genome, to characterize whether the MHC appeared unusual in this regard. Finally, we used the Spearman’s rank correlation coefficient *ρ* and similarity scores *SC* to investigate fine scale evidence of non-random mating at individual SNPs, classical HLA genes, and amino acids.

### 3.1. Whole MHC Region

Following confirmation that individuals in the HRS dataset were of European ancestry (Figure S1), we plotted *R* across the MHC region against genome-wide SNP relatedness (excluding chromosome 6) as calculated by the GCTA software for spousal and non-spouse pairs (Figure S2). We found little evidence to suggest that spousal pairs who were most genetically similar at the MHC were also most genetically similar across the genome. Regressing spousal similarity at the MHC on genome-wide similarity was not significant (*p* > 0.05). In addition, there were no outlying pairs that showed extreme genome-wide similarity/dissimilarity. These results suggest that spousal similarity induced by population stratification is unlikely to mask any MHC dissimilarity induced by disassortative mating in our dataset. 

**Figure 1:**
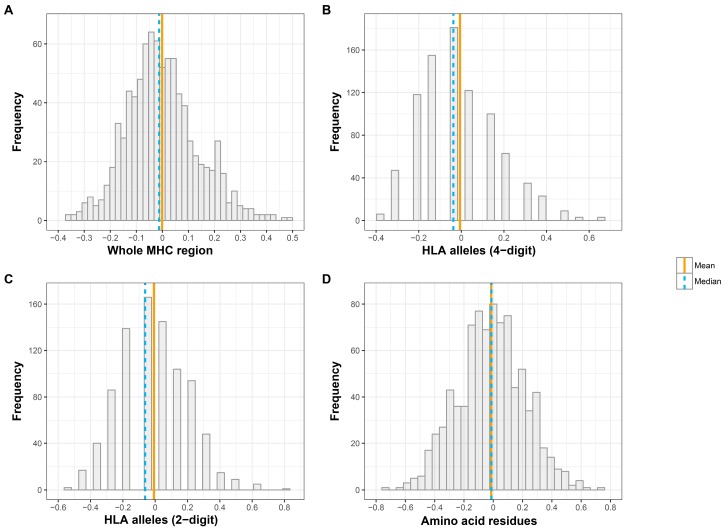
Distribution of relatedness coefficients (x axis) across the Major Histocompatibility Complex (MHC) region among spousal pairs. (**A**) all single-nucleotide polymorphisms (SNPs), (**B**) all eight classical human leukocyte antigen (HLA) genes (at four-digit resolution), (**C**) all eight classical HLA genes (at two-digit resolution), and (**D**) all 376 amino acid polymorphisms.

The distribution of *R* between spouses at three different levels of genomic variation is displayed in Figure 1. Using SNP data, the *R* coefficient showed a slight positive-skewed distribution, suggesting that the median might be a better measure of central tendency than the mean for this measure. The distribution of *R* across spouse pairs was slightly positively skewed in the case of four- and two-digit classical HLA alleles also. In contrast, the *R* coefficient approached a normal distribution in the case of amino acids.

The results of relatedness analyses at three levels of genomic variation are summarized in Table 1. Spouses display slight dissimilarity at all of the three levels of genomic variation, but these do not reach statistical significance. Using molecular markers (average relatedness coefficients across 4678 SNPs), we observed that the median MHC relatedness between spouses in the HRS dataset at the MHC loci was comparable to randomly selected opposite-sex pairs (*R_median(spouse)_* = −0.012, two-tailed *p* = 0.829). Second, based on HLA types (average relatedness coefficients across eight classical HLA genes), no significant MHC-dissimilarity was observed among spouses at either four- or two-digit classifications. Lastly, in the case of the multilocus relatedness coefficients based on the amino acid data, the actual couples were significantly dissimilar relative to random pairs (*R_mean(spouse)_* = −0.014, two-tailed *p*-value = 0.040); however, the significance disappeared when we looked at the median MHC relatedness between spouses. 

**Figure 2:**
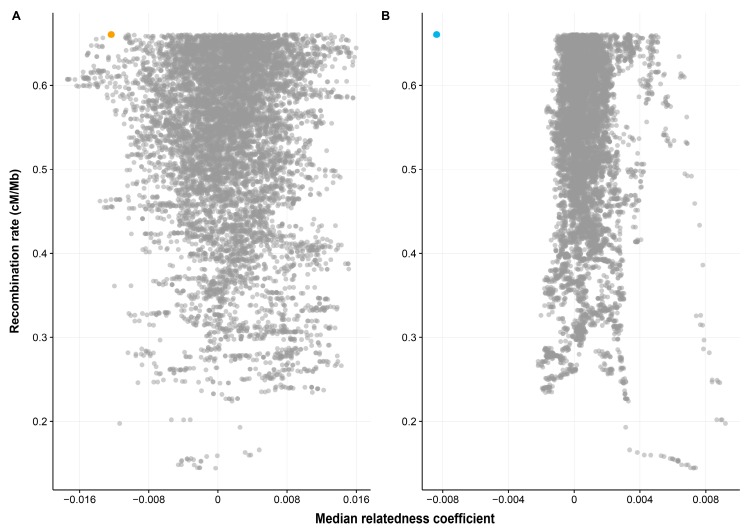
Median relatedness coefficients for the sampled 4.97 Mb genomic regions (*N* = 10,000) across the genome between (**A**) spouses (**B**) opposite-sex non-spouse pairs. Each grey dot represents one sampled genomic region. The MHC region for spouses and non-spouse pairs were plotted in yellow (in **A**) and blue (in **B**), respectively. The outlying points in the right hand side of panel **B** represent overlapping windows from the same genomic region of chromosome 3p- an area of particularly high linkage disequilibrium and low recombination.

For the genomic windows analyses, only 0.81% of the 10,000 randomly sampled genomic windows exhibited more extreme dissimilarity than the MHC region among spousal pairs (Figure 2A). However, this pattern was repeated and even more extreme among opposite-sex non-spouse pairs (Figure 2B). It should be noted that since the number of spouses (872 pairs) is much smaller than the number of non-spousal pairs (759,512 pairs), there is a larger standard error of the median relatedness of the sample genomic windows between spouses and consequently much tighter clustering of the related coefficients around the null among the non-spouse pairs. Our result strongly suggests that the relatedness pattern at the MHC level is extreme when compared to the rest of the genome, and that the MHC dissimilarity in spouses relative to the rest of the genome is as extreme as it is in opposite-sex non-spouse pairs. 

### 3.2. Individual SNPs, HLA Alleles, and Amino Acids 

Further analyses were conducted to identify potential individual SNPs, HLA genes, and amino acid residues within the MHC region exhibiting non-random mating patterns. 

**Figure 3:**
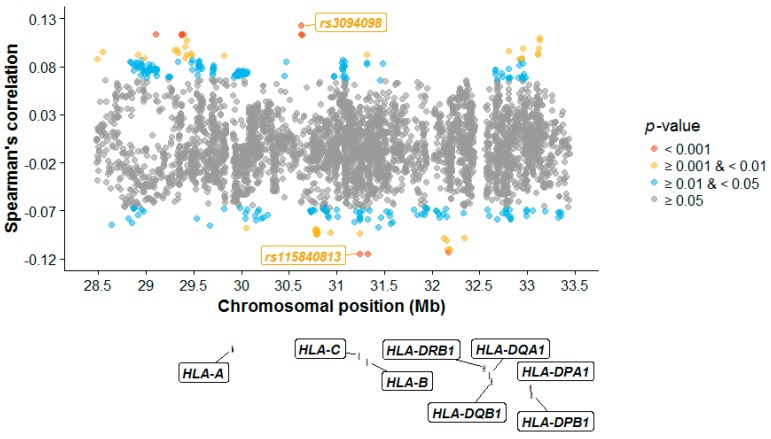
MHC correlation plot. The Spearman’s rank correlation coefficient (*ρ*) between spouses at each SNP is plotted against its physical chromosomal position. All nominally significant SNPs (based on two-sided *p*-values and before correction for multiple comparisons) are plotted in colour. The positions of the classical HLA genes are from UCSC (GRCh37/hg19) and superimposed in the plot.

Using Spearman’s rank correlation coefficient *ρ*, no individual SNP remained significant after multiple correction (Figure 3; Table S4). The top MHC SNP *rs3094098* (*ρ* = 0.123, two-sided *p*-value = 0.0003) is located in the DEAH-Box Helicase 16 (*DHX16*) gene and shows similarity between spouses [41]. *DHX16* encodes DEAD box proteins which are putative RNA helicases and are implicated in cell cycle progression [42]. Single-nucleotide polymorphism *rs115840813* was the SNP showing the most amount of dissimilarity (*ρ* = −0.115, two-sided *p*-value = 0.0007). It is a non-coding transcript exon variant, located in a processed pseudogene, *USP8P1*.

In the case of classical HLA genes, we conducted analyses for both four- and two-digit classification and found similar results; that is, although several HLA genes exhibited below average similarity, no significant nominal *p*-value was observed (Table 2; Table S5). 

**Figure 4:**
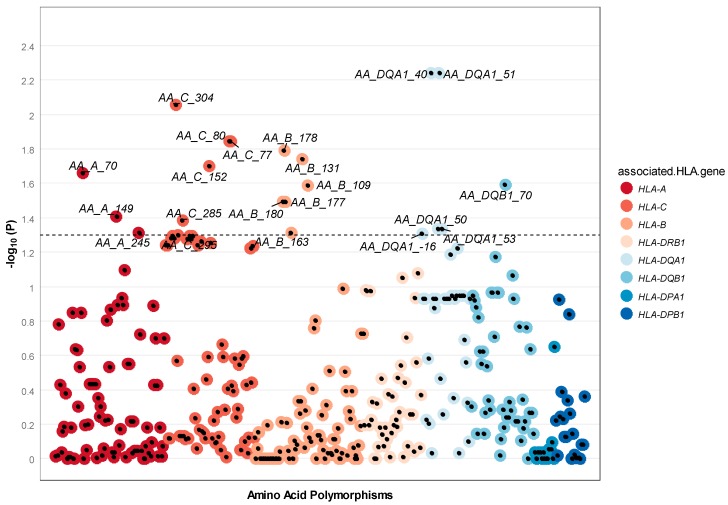
Manhattan two-sided *p*-value for each amino acid polymorphism tested. HLA Amino acid polymorphisms are displayed along the *X*-axis, with minus log ten two-sided *p*-value displayed on the *Y*-axis. Each data point in the plot signifies an amino acid residue. The color of the data point indicates the different HLA genes.

In this analysis, we tested both amino acids within the protein structure and in the signal peptide (i.e., the short peptide present at the N-terminus of the nascent HLA precursor protein denoted by negative amino acid positions). Using the similarity score *SC*, 21 amino acid residues from the HLA region showed nominal dissimilarity among spouses (Figure 4); however, no individual amino acid polymorphism remained significant after correction for multiple testing (Table S6). The top signal was found for amino acid position 40 and 51 in *HLA-DQA1* (two-sided *p*-value = 0.006).

## 4. Discussion

In this study we performed a set of analyses to investigate genetic signatures of non-random mating patterns in 872 European American spousal pairs from the HRS dataset. We found no strong evidence to support a role for the MHC region in human mate selection. Neither the MHC region, or any single SNPs, classic HLA alleles, or amino acid polymorphisms within the MHC region, were significantly dissimilar between spouses relative to non-spouse pairs. Whilst genetic dissimilarity between spouses at the MHC was extreme compared to the rest of the genome, this same pattern was also observed in random pairings of opposite-sex individuals.

### 4.1. Dissimilarity between Spouses across the MHC Region as a Whole

There has been considerable debate in the literature regarding the existence of MHC-dependent mate selection [6,8,9,10,43]. Using a similar definition of the relatedness coefficient *R* as used in this work, Chaix et al. measured genetic similarity across 9010 SNPs spread throughout the MHC region for 28 European American couples in the HapMap phase 2 dataset, and found that mating pairs were significantly more MHC-dissimilar than random opposite-sex pairs of individuals [6]. However, Derti et al. did not find any evidence for disassortative mating in the HapMap phase 3 dataset, and noted that minor changes to the methods of Chaix et al. would decrease the significance, or have rendered the original results from the HapMap 2 dataset non-significant [8]. Such modifications included increasing the number of permutations, removing the couple displaying the highest MHC dissimilarity, or using the median rather than mean relatedness to quantify the MHC dissimilarity among spouses, etc. When Derti et al. examined 24 couples who were only present in the HapMap Phase 3 dataset; virtually no evidence for disassortative mating across the MHC was found (*R_mean(spouses)_* = −0.014, two-sided *p*-value = 0.351). Since the couples selected from Phase 2 and 3 of the HapMap were from the same population (and therefore free of population stratification) and highly concordant in terms of data quality, the authors concluded that the findings in the previous study were likely driven by sporadic outliers.

Similar to Derti et al., by treating the entire MHC locus as a single entity, we did not find any strong evidence to support a role for the MHC in mate selection. Although there was some nominal evidence of dissimilarity between couples at classical HLA alleles based on two-digit resolution, the effect was marginal and disappeared when we examined median rather than mean levels of allele sharing. We consider our results robust for the following reasons. First, our results do not appear to be driven by spousal pairs with extreme genetic (dis)similarity at the MHC (Figure 1; Figure S3). Second, we took steps to ensure that our results were not driven by latent population stratification. All individuals were broadly of European ancestry (Figure S1). Third, we showed that genomic relatedness between spousal pairs across the MHC was not correlated with genetic relatedness across the rest of the genome (Figure S2). If fine-scale population substructure were masking disassortment at the MHC, we would expect genetic sharing between spouses at the MHC to be correlated with genetic similarity between spouses across the genome. This result suggests that population stratification is unlikely to be a major contributor to spousal similarity at the MHC in our dataset. Fourth, we vastly increased the number of spousal pairs (~900 pairs) compared to previous studies of disassortative mating in the MHC. Finally, we increased the number of permutations run to evaluate significance compared to previous studies to 100,000, which reduced the margin of error around our estimates of the permuted *p*-value [44].

### 4.2. Dissimilarity between Spouses at Individual SNPs, HLA Alleles, and Amino Acids

Given the above results across the entirety of the MHC, we expected the likelihood that any individual effects at finer scales would be subtle in our sample, and therefore we were not surprised to find no individual SNPs, classical HLA genes, or amino acids were significant after adjustment for multiple comparisons. Similar to our work, Laurent et al. performed a genome-wide scan to detect genes potentially involved in mating choices, and found none of the classical HLA genes were significant in HapMap phase 3 European American couples [45]. In contrast, Khankhanian et al. reported significant similarity at MHC class I region, as well as significant dissimilarity at two classical HLA genes within the MHC class II region (i.e., *HLA-DQA1* and *HLA-DQB1*), among 930 spouses in a European and European American sample [32]. However, this dataset consisted of couples having children afflicted with multiple sclerosis, a disease showing strong association with the MHC region. It is likely that ascertaining spouses who have a child with multiple sclerosis may lead to a spurious correlation between parental risk alleles at the MHC (i.e., collider bias), and so findings from this study should be treated with caution.

### 4.3. General Discussion

Reports of negative assortment at MHC loci in the literature have been inconsistent, although there is some evidence to suggest that MHC-dependent mate selection in humans may be population-specific [6,25,27,28,29,30]. For example, two previous studies supporting the existence of MHC-related disassortative mating were both based on cultural isolates: the Hutterites [30] and the Mormons [6]. This has led to speculation that in population isolates, selection on the MHC might play a role in avoidance of inbreeding, rather than disassortative mating per se. However, effects such as these, if they exist, are likely to be small. There is no evidence for negative assortment in the MHC in population groups that experience high pathogen pressure, such as Amerindian tribes [28], in which, presumably, the preference for mating with an individual carrying particular pathogen-resistant alleles is far more important than mating with MHC-dissimilar individuals. Additional factors that might explain some of the discrepancies in results between studies involve differences in generational and social factors between the different cohorts. For example, the individuals in the present study (mainly born in the 1930s and 1940s) would have chosen spousal partners at a time when such choices may have been influenced by geographical and social endogamy. These considerations may have served to obscure the effect of biological factors in mate choice.

## 5. Conclusions

In summary, despite long-standing hypotheses and a smattering of smaller studies relating the MHC to mate choice in humans, we found no significant differences in MHC dissimilarity between spouses and non-spouse pairs at either genotypic, allelic, or amino acid levels in HRS European Americans. Mate selection in many modern human populations is no doubt a complex decision process depending on biological, psychological, and socio-cultural factors. Our results suggest that the relative importance of the MHC in making these decisions appears to be negligible.

## Figures and Tables

**Table 1:** Summary of relatedness analyses across the entire MHC region calculated across three levels of genomic variation (SNPs, classical HLA genes, and amino acid polymorphisms) in the Health and Retirement Study (HRS) individuals of European ancestry.

Datasets	Parameters	Relatedness ^1^	*p*-Value ^2^
SNPs	Mean ^3^	−0.001 ± 0.137	0.829
	Median ^4^	−0.012 ± 0.124	0.218
Classical HLA genes (four-digit resolution)	Mean	−0.007 ± 0.184	0.258
	Median	−0.037 ± 0.128	0.500
Classical HLA genes (two-digit resolution)	Mean	−0.008 ± 0.209	0.211
	Median	−0.062 ± 0.143	0.419
Amino acids	Mean	−0.014 ± 0.226	0.040
	Median	−0.013 ± 0.214	0.235

^1^ Descriptive statistics of the relatedness coefficients between real couples (*N* = 872); ^2^ two-sided *p*-value, derived from the 100,000 permutations; ^3^ the mean relatedness coefficient (±1 SD) across all real couples; SD: standard deviation; ^4^ the median relatedness coefficient (±1 MAD) across all real couples; MAD: median absolute deviation, MAD = (median|x-median(x)|)/0.6745.

**Table 2:** Summary of similarity scores (*SC*) for each HLA gene (at four-digit resolution) in HRS European Americans.

HLA Gene	Similarity Score (*SC*) ^1^	Similarity Score (*SC*) ^2^		Two-Sided *p*-Value
		Mean	SD	
*A*	411	421.90	14.37	0.448
*C*	260	285.20	13.98	0.071
*B*	191	207.78	12.31	0.173
*DRB1*	259	265.61	13.49	0.624
*DQA1*	488	497.30	16.47	0.572
*DQB1*	324	331.61	14.85	0.608
*DPA1*	1275	1267.73	11.95	0.543
*DPB1*	585	586.24	13.72	0.928

^1^ Similarity scores between spouses; ^2^ similarity scores summarized from normal distribution.

## Supplementary Materials

The following are available online at http://www.mdpi.com/2073-4425/9/1/53/s1, Figure S1: Ancestry clustering based on genome-wide association data, A: plot showing the first and second principal components (i.e., PC1 vs. PC2); B: plot showing the second and third principal components (i.e., PC2 vs. PC3); C: plot showing the third and fourth principal components (i.e., PC3 vs. PC4). 1000 Genomes Project reference individuals: EUR (European- steel blue), AFR (African- dark red), EAS (East Asian- sea green), SAS (South Asian- firebrick), AMR (Admixed American- orange), HRS (Health and Retirement Study) samples: light blue. Figure S2: Scatter plot of the relatedness coefficients *R* across the MHC region calculated using genotype data and genome-wide SNP relatedness (excludes chromosome 6) as calculated by the GCTA software. Red and yellow dots correspond to spousal and non-spouse pairs, respectively. Figure S3: Distribution of relatedness coefficients (x axis) across the MHC region among random opposite-sex non-spousal pairs, A: all SNPs; B: all eight classical HLA genes (at four-digit resolution); C: all eight classical HLA genes (at two-digit resolution); D: all 376 amino acid polymorphisms. Table S1: The count and percentage of individuals having at least one missing allele at classical HLA loci (four-digit resolution). Table S2: The count and percentage of individuals having at least one missing allele at classical HLA loci (two-digit resolution). Table S3: The count and percentage of individuals having at least one missing allele at each amino acid residue. Table S4: Summary of Spearman’s rank correlation coefficients (*ρ*) for each SNP in HRS European Americans. Table S5: Summary of similarity scores (*SC*) for each HLA gene (at two-digit resolution) in HRS European Americans. Table S6: Summary of similarity scores (*SC*) for each HLA amino acid residue in HRS European Americans.
