# Supplementary material for: MHC-Dependent Mate Selection within 872 Spousal Pairs of European Ancestry from the Health and Retirement Study
Source: Genes (Basel). 2018 Jan 22;9(1):53. doi: 10.3390/genes9010053 (PMC5793204; doi:10.3390/genes9010053)
Supplement: Supplementary file 1 [file genes-09-00053-s001.zip › Table S1-3.docx]

| HLA alleles (4-digit resolution) | Counts | Missing rate |
| --- | --- | --- |
| *A* | 2 | 0.023% |
| *C* | 7 | 0.081% |
| *B* | 13 | 0.150% |
| *DRB1* | 6 | 0.069% |
| *DQA1* | 4 | 0.046% |
| *DQB1* | 17 | 0.196% |
| *DPA1* | 10 | 0.116% |
| *DPB1* | 13 | 0.150% |

**Table S1.** The count and percentage of individuals having at least one missing allele at classical HLA loci (4-digit resolution).

**Table S2.** The count and percentage of individuals having at least one missing allele at classical HLA loci (2-digit resolution).

| HLA alleles (2-digit resolution) | Counts | Missing rate |
| --- | --- | --- |
| *A* | 4 | 0.046% |
| *C* | 4 | 0.046% |
| *B* | 6 | 0.069% |
| *DRB1* | 2 | 0.023% |
| *DQA1* | 0 | 0.000% |
| *DQB1* | 4 | 0.046% |
| *DPA1* | 3 | 0.035% |
| *DPB1* | 9 | 0.104% |

**Table S3.** The count and percentage of individuals having at least one missing allele at each amino acid residue.

| HLA amino acid residues | Counts | Missing rate |
| --- | --- | --- |
| *AA_A_-22* | 13 | 0.150% |
| *AA_A_-20* | 13 | 0.150% |
| *AA_A_-15* | 14 | 0.162% |
| *AA_A_-11* | 13 | 0.150% |
| *AA_A_-2* | 13 | 0.150% |
| *AA_A_9* | 0 | 0.000% |
| *AA_A_12* | 0 | 0.000% |
| *AA_A_17* | 0 | 0.000% |
| *AA_A_19* | 0 | 0.000% |
| *AA_A_31* | 0 | 0.000% |
| *AA_A_35* | 0 | 0.000% |
| *AA_A_43* | 0 | 0.000% |
| *AA_A_44* | 0 | 0.000% |
| *AA_A_56* | 0 | 0.000% |
| *AA_A_62* | 0 | 0.000% |
| *AA_A_63* | 1 | 0.012% |
| *AA_A_65* | 0 | 0.000% |
| *AA_A_66* | 0 | 0.000% |
| *AA_A_67* | 0 | 0.000% |
| *AA_A_70* | 0 | 0.000% |
| *AA_A_73* | 1 | 0.012% |
| *AA_A_74* | 1 | 0.012% |
| *AA_A_76* | 2 | 0.023% |
| *AA_A_77* | 2 | 0.023% |
| *AA_A_79* | 0 | 0.000% |
| *AA_A_80* | 0 | 0.000% |
| *AA_A_81* | 0 | 0.000% |
| *AA_A_82* | 0 | 0.000% |
| *AA_A_83* | 0 | 0.000% |
| *AA_A_90* | 0 | 0.000% |
| *AA_A_95* | 2 | 0.023% |
| *AA_A_97* | 2 | 0.023% |
| *AA_A_99* | 1 | 0.012% |
| *AA_A_102* | 0 | 0.000% |
| *AA_A_105* | 0 | 0.000% |
| *AA_A_107* | 0 | 0.000% |
| *AA_A_109* | 0 | 0.000% |
| *AA_A_114* | 4 | 0.046% |
| *AA_A_116* | 2 | 0.023% |
| *AA_A_127* | 0 | 0.000% |
| *AA_A_142* | 0 | 0.000% |
| *AA_A_144* | 1 | 0.012% |
| *AA_A_145* | 0 | 0.000% |
| *AA_A_149* | 0 | 0.000% |
| *AA_A_150* | 0 | 0.000% |
| *AA_A_151* | 0 | 0.000% |
| *AA_A_152* | 2 | 0.023% |
| *AA_A_156* | 1 | 0.012% |
| *AA_A_158* | 0 | 0.000% |
| *AA_A_161* | 0 | 0.000% |
| *AA_A_163* | 2 | 0.023% |
| *AA_A_166* | 0 | 0.000% |
| *AA_A_167* | 0 | 0.000% |
| *AA_A_171* | 0 | 0.000% |
| *AA_A_184* | 2 | 0.023% |
| *AA_A_186* | 1 | 0.012% |
| *AA_A_193* | 2 | 0.023% |
| *AA_A_194* | 2 | 0.023% |
| *AA_A_207* | 1 | 0.012% |
| *AA_A_245* | 2 | 0.023% |
| *AA_A_246* | 1 | 0.012% |
| *AA_A_253* | 2 | 0.023% |
| *AA_A_255* | 1 | 0.012% |
| *AA_A_268* | 1 | 0.012% |
| *AA_A_276* | 14 | 0.162% |
| *AA_A_282* | 14 | 0.162% |
| *AA_A_283* | 14 | 0.162% |
| *AA_A_288* | 14 | 0.162% |
| *AA_A_294* | 14 | 0.162% |
| *AA_A_297* | 14 | 0.162% |
| *AA_A_298* | 14 | 0.162% |
| *AA_A_299* | 13 | 0.150% |
| *AA_A_307* | 13 | 0.150% |
| *AA_A_310* | 14 | 0.162% |
| *AA_A_311* | 13 | 0.150% |
| *AA_A_314* | 14 | 0.162% |
| *AA_A_321* | 14 | 0.162% |
| *AA_A_334* | 12 | 0.139% |
| *AA_C_339* | 6 | 0.069% |
| *AA_C_326* | 6 | 0.069% |
| *AA_C_309* | 6 | 0.069% |
| *AA_C_308* | 6 | 0.069% |
| *AA_C_307* | 8 | 0.092% |
| *AA_C_306* | 8 | 0.092% |
| *AA_C_305* | 8 | 0.092% |
| *AA_C_304* | 10 | 0.116% |
| *AA_C_303* | 11 | 0.127% |
| *AA_C_295* | 11 | 0.127% |
| *AA_C_291* | 9 | 0.104% |
| *AA_C_289* | 9 | 0.104% |
| *AA_C_285* | 11 | 0.127% |
| *AA_C_284* | 9 | 0.104% |
| *AA_C_275* | 16 | 0.185% |
| *AA_C_273* | 3 | 0.035% |
| *AA_C_270* | 1 | 0.012% |
| *AA_C_267* | 3 | 0.035% |
| *AA_C_261* | 3 | 0.035% |
| *AA_C_253* | 3 | 0.035% |
| *AA_C_248* | 1 | 0.012% |
| *AA_C_219* | 7 | 0.081% |
| *AA_C_211* | 1 | 0.012% |
| *AA_C_194* | 2 | 0.023% |
| *AA_C_193* | 1 | 0.012% |
| *AA_C_184* | 2 | 0.023% |
| *AA_C_177* | 0 | 0.000% |
| *AA_C_173* | 0 | 0.000% |
| *AA_C_170* | 0 | 0.000% |
| *AA_C_163* | 1 | 0.012% |
| *AA_C_156* | 2 | 0.023% |
| *AA_C_152* | 1 | 0.012% |
| *AA_C_147* | 0 | 0.000% |
| *AA_C_143* | 0 | 0.000% |
| *AA_C_138* | 0 | 0.000% |
| *AA_C_116* | 16 | 0.185% |
| *AA_C_114* | 1 | 0.012% |
| *AA_C_113* | 0 | 0.000% |
| *AA_C_103* | 0 | 0.000% |
| *AA_C_99* | 3 | 0.035% |
| *AA_C_97* | 1 | 0.012% |
| *AA_C_95* | 2 | 0.023% |
| *AA_C_94* | 0 | 0.000% |
| *AA_C_91* | 0 | 0.000% |
| *AA_C_90* | 0 | 0.000% |
| *AA_C_80* | 0 | 0.000% |
| *AA_C_77* | 0 | 0.000% |
| *AA_C_73* | 0 | 0.000% |
| *AA_C_66* | 0 | 0.000% |
| *AA_C_49* | 0 | 0.000% |
| *AA_C_35* | 0 | 0.000% |
| *AA_C_24* | 0 | 0.000% |
| *AA_C_21* | 0 | 0.000% |
| *AA_C_16* | 0 | 0.000% |
| *AA_C_14* | 0 | 0.000% |
| *AA_C_11* | 0 | 0.000% |
| *AA_C_9* | 2 | 0.023% |
| *AA_C_6* | 0 | 0.000% |
| *AA_C_1* | 7 | 0.081% |
| *AA_C_-5* | 7 | 0.081% |
| *AA_C_-9* | 7 | 0.081% |
| *AA_C_-15* | 8 | 0.092% |
| *AA_C_-17* | 8 | 0.092% |
| *AA_C_-18* | 8 | 0.092% |
| *AA_B_325* | 20 | 0.231% |
| *AA_B_307* | 1 | 0.012% |
| *AA_B_305* | 3 | 0.035% |
| *AA_B_300* | 1 | 0.012% |
| *AA_B_299* | 1 | 0.012% |
| *AA_B_298* | 1 | 0.012% |
| *AA_B_296* | 1 | 0.012% |
| *AA_B_295* | 1 | 0.012% |
| *AA_B_282* | 5 | 0.058% |
| *AA_B_275* | 1 | 0.012% |
| *AA_B_270* | 0 | 0.000% |
| *AA_B_268* | 0 | 0.000% |
| *AA_B_267* | 0 | 0.000% |
| *AA_B_253* | 0 | 0.000% |
| *AA_B_245* | 0 | 0.000% |
| *AA_B_239* | 0 | 0.000% |
| *AA_B_211* | 0 | 0.000% |
| *AA_B_199* | 1 | 0.012% |
| *AA_B_194* | 1 | 0.012% |
| *AA_B_180* | 0 | 0.000% |
| *AA_B_178* | 0 | 0.000% |
| *AA_B_177* | 0 | 0.000% |
| *AA_B_171* | 0 | 0.000% |
| *AA_B_167* | 0 | 0.000% |
| *AA_B_166* | 0 | 0.000% |
| *AA_B_163* | 4 | 0.046% |
| *AA_B_162* | 0 | 0.000% |
| *AA_B_158* | 1 | 0.012% |
| *AA_B_156* | 2 | 0.023% |
| *AA_B_152* | 0 | 0.000% |
| *AA_B_147* | 0 | 0.000% |
| *AA_B_145* | 0 | 0.000% |
| *AA_B_143* | 0 | 0.000% |
| *AA_B_131* | 0 | 0.000% |
| *AA_B_116* | 16 | 0.185% |
| *AA_B_114* | 5 | 0.058% |
| *AA_B_113* | 1 | 0.012% |
| *AA_B_109* | 0 | 0.000% |
| *AA_B_103* | 0 | 0.000% |
| *AA_B_99* | 0 | 0.000% |
| *AA_B_97* | 2 | 0.023% |
| *AA_B_95* | 0 | 0.000% |
| *AA_B_94* | 0 | 0.000% |
| *AA_B_90* | 0 | 0.000% |
| *AA_B_83* | 0 | 0.000% |
| *AA_B_82* | 0 | 0.000% |
| *AA_B_81* | 0 | 0.000% |
| *AA_B_80* | 3 | 0.035% |
| *AA_B_77* | 2 | 0.023% |
| *AA_B_76* | 0 | 0.000% |
| *AA_B_74* | 0 | 0.000% |
| *AA_B_71* | 0 | 0.000% |
| *AA_B_70* | 0 | 0.000% |
| *AA_B_69* | 0 | 0.000% |
| *AA_B_67* | 3 | 0.035% |
| *AA_B_66* | 0 | 0.000% |
| *AA_B_65* | 0 | 0.000% |
| *AA_B_63* | 0 | 0.000% |
| *AA_B_62* | 0 | 0.000% |
| *AA_B_59* | 0 | 0.000% |
| *AA_B_52* | 0 | 0.000% |
| *AA_B_46* | 0 | 0.000% |
| *AA_B_45* | 2 | 0.023% |
| *AA_B_41* | 0 | 0.000% |
| *AA_B_32* | 0 | 0.000% |
| *AA_B_30* | 0 | 0.000% |
| *AA_B_24* | 0 | 0.000% |
| *AA_B_12* | 0 | 0.000% |
| *AA_B_11* | 0 | 0.000% |
| *AA_B_9* | 0 | 0.000% |
| *AA_B_4* | 0 | 0.000% |
| *AA_B_-8* | 0 | 0.000% |
| *AA_B_-10* | 0 | 0.000% |
| *AA_B_-11* | 0 | 0.000% |
| *AA_B_-16* | 0 | 0.000% |
| *AA_B_-21* | 0 | 0.000% |
| *AA_B_-23* | 0 | 0.000% |
| *AA_DRB1_233* | 632 | 7.305% |
| *AA_DRB1_231* | 631 | 7.293% |
| *AA_DRB1_189* | 632 | 7.305% |
| *AA_DRB1_181* | 416 | 4.808% |
| *AA_DRB1_180* | 416 | 4.808% |
| *AA_DRB1_166* | 3 | 0.035% |
| *AA_DRB1_149* | 4 | 0.046% |
| *AA_DRB1_142* | 3 | 0.035% |
| *AA_DRB1_140* | 4 | 0.046% |
| *AA_DRB1_133* | 3 | 0.035% |
| *AA_DRB1_120* | 4 | 0.046% |
| *AA_DRB1_112* | 4 | 0.046% |
| *AA_DRB1_104* | 4 | 0.046% |
| *AA_DRB1_98* | 4 | 0.046% |
| *AA_DRB1_96* | 3 | 0.035% |
| *AA_DRB1_86* | 0 | 0.000% |
| *AA_DRB1_85* | 0 | 0.000% |
| *AA_DRB1_78* | 0 | 0.000% |
| *AA_DRB1_77* | 0 | 0.000% |
| *AA_DRB1_74* | 5 | 0.058% |
| *AA_DRB1_73* | 0 | 0.000% |
| *AA_DRB1_71* | 16 | 0.185% |
| *AA_DRB1_70* | 4 | 0.046% |
| *AA_DRB1_67* | 7 | 0.081% |
| *AA_DRB1_60* | 3 | 0.035% |
| *AA_DRB1_58* | 2 | 0.023% |
| *AA_DRB1_57* | 5 | 0.058% |
| *AA_DRB1_47* | 0 | 0.000% |
| *AA_DRB1_40* | 0 | 0.000% |
| *AA_DRB1_38* | 2 | 0.023% |
| *AA_DRB1_37* | 4 | 0.046% |
| *AA_DRB1_33* | 0 | 0.000% |
| *AA_DRB1_32* | 0 | 0.000% |
| *AA_DRB1_31* | 0 | 0.000% |
| *AA_DRB1_30* | 0 | 0.000% |
| *AA_DRB1_28* | 1 | 0.012% |
| *AA_DRB1_26* | 21 | 0.243% |
| *AA_DRB1_25* | 0 | 0.000% |
| *AA_DRB1_16* | 0 | 0.000% |
| *AA_DRB1_14* | 0 | 0.000% |
| *AA_DRB1_13* | 1 | 0.012% |
| *AA_DRB1_12* | 0 | 0.000% |
| *AA_DRB1_11* | 0 | 0.000% |
| *AA_DRB1_10* | 1 | 0.012% |
| *AA_DRB1_9* | 0 | 0.000% |
| *AA_DRB1_4* | 16 | 0.185% |
| *AA_DRB1_-1* | 679 | 7.848% |
| *AA_DRB1_-16* | 679 | 7.848% |
| *AA_DRB1_-17* | 679 | 7.848% |
| *AA_DRB1_-24* | 679 | 7.848% |
| *AA_DRB1_-25* | 679 | 7.848% |
| *AA_DQA1_-16* | 0 | 0.000% |
| *AA_DQA1_11* | 0 | 0.000% |
| *AA_DQA1_18* | 0 | 0.000% |
| *AA_DQA1_25* | 0 | 0.000% |
| *AA_DQA1_26* | 0 | 0.000% |
| *AA_DQA1_34* | 0 | 0.000% |
| *AA_DQA1_40* | 0 | 0.000% |
| *AA_DQA1_41* | 0 | 0.000% |
| *AA_DQA1_45* | 0 | 0.000% |
| *AA_DQA1_47* | 0 | 0.000% |
| *AA_DQA1_48* | 0 | 0.000% |
| *AA_DQA1_50* | 0 | 0.000% |
| *AA_DQA1_51* | 0 | 0.000% |
| *AA_DQA1_52* | 1 | 0.012% |
| *AA_DQA1_53* | 1 | 0.012% |
| *AA_DQA1_54* | 0 | 0.000% |
| *AA_DQA1_55* | 0 | 0.000% |
| *AA_DQA1_56* | 5532 | 63.939% |
| *AA_DQA1_61* | 0 | 0.000% |
| *AA_DQA1_64* | 0 | 0.000% |
| *AA_DQA1_66* | 0 | 0.000% |
| *AA_DQA1_69* | 1 | 0.012% |
| *AA_DQA1_75* | 0 | 0.000% |
| *AA_DQA1_76* | 1 | 0.012% |
| *AA_DQA1_80* | 0 | 0.000% |
| *AA_DQA1_107* | 3 | 0.035% |
| *AA_DQA1_129* | 1 | 0.012% |
| *AA_DQA1_130* | 0 | 0.000% |
| *AA_DQA1_156* | 1 | 0.012% |
| *AA_DQA1_161* | 1 | 0.012% |
| *AA_DQA1_163* | 1 | 0.012% |
| *AA_DQA1_175* | 0 | 0.000% |
| *AA_DQA1_187* | 2 | 0.023% |
| *AA_DQA1_207* | 4 | 0.046% |
| *AA_DQA1_215* | 4 | 0.046% |
| *AA_DQA1_218* | 4 | 0.046% |
| *AA_DQB1_224* | 19 | 0.220% |
| *AA_DQB1_221* | 19 | 0.220% |
| *AA_DQB1_220* | 38 | 0.439% |
| *AA_DQB1_203* | 35 | 0.405% |
| *AA_DQB1_197* | 18 | 0.208% |
| *AA_DQB1_185* | 18 | 0.208% |
| *AA_DQB1_182* | 18 | 0.208% |
| *AA_DQB1_167* | 18 | 0.208% |
| *AA_DQB1_140* | 18 | 0.208% |
| *AA_DQB1_135* | 18 | 0.208% |
| *AA_DQB1_130* | 18 | 0.208% |
| *AA_DQB1_126* | 18 | 0.208% |
| *AA_DQB1_125* | 18 | 0.208% |
| *AA_DQB1_116* | 18 | 0.208% |
| *AA_DQB1_90* | 19 | 0.220% |
| *AA_DQB1_89* | 17 | 0.196% |
| *AA_DQB1_87* | 18 | 0.208% |
| *AA_DQB1_86* | 17 | 0.196% |
| *AA_DQB1_85* | 17 | 0.196% |
| *AA_DQB1_84* | 17 | 0.196% |
| *AA_DQB1_77* | 0 | 0.000% |
| *AA_DQB1_75* | 0 | 0.000% |
| *AA_DQB1_74* | 0 | 0.000% |
| *AA_DQB1_71* | 0 | 0.000% |
| *AA_DQB1_70* | 0 | 0.000% |
| *AA_DQB1_67* | 0 | 0.000% |
| *AA_DQB1_66* | 0 | 0.000% |
| *AA_DQB1_57* | 2 | 0.023% |
| *AA_DQB1_56* | 0 | 0.000% |
| *AA_DQB1_55* | 1 | 0.012% |
| *AA_DQB1_53* | 0 | 0.000% |
| *AA_DQB1_52* | 0 | 0.000% |
| *AA_DQB1_47* | 0 | 0.000% |
| *AA_DQB1_46* | 0 | 0.000% |
| *AA_DQB1_45* | 0 | 0.000% |
| *AA_DQB1_38* | 0 | 0.000% |
| *AA_DQB1_37* | 2 | 0.023% |
| *AA_DQB1_30* | 2 | 0.023% |
| *AA_DQB1_28* | 0 | 0.000% |
| *AA_DQB1_26* | 1 | 0.012% |
| *AA_DQB1_23* | 0 | 0.000% |
| *AA_DQB1_14* | 1 | 0.012% |
| *AA_DQB1_13* | 1 | 0.012% |
| *AA_DQB1_9* | 1 | 0.012% |
| *AA_DQB1_3* | 18 | 0.208% |
| *AA_DQB1_-4* | 636 | 7.351% |
| *AA_DQB1_-5* | 638 | 7.374% |
| *AA_DQB1_-6* | 636 | 7.351% |
| *AA_DQB1_-9* | 637 | 7.362% |
| *AA_DQB1_-10* | 636 | 7.351% |
| *AA_DQB1_-17* | 636 | 7.351% |
| *AA_DQB1_-18* | 636 | 7.351% |
| *AA_DQB1_-21* | 639 | 7.386% |
| *AA_DQB1_-27* | 637 | 7.362% |
| *AA_DPA1_228* | 21 | 0.243% |
| *AA_DPA1_190* | 20 | 0.231% |
| *AA_DPA1_160* | 21 | 0.243% |
| *AA_DPA1_127* | 20 | 0.231% |
| *AA_DPA1_111* | 21 | 0.243% |
| *AA_DPA1_96* | 20 | 0.231% |
| *AA_DPA1_83* | 0 | 0.000% |
| *AA_DPA1_73* | 0 | 0.000% |
| *AA_DPA1_72* | 0 | 0.000% |
| *AA_DPA1_66* | 0 | 0.000% |
| *AA_DPA1_50* | 0 | 0.000% |
| *AA_DPA1_31* | 0 | 0.000% |
| *AA_DPA1_28* | 0 | 0.000% |
| *AA_DPA1_18* | 0 | 0.000% |
| *AA_DPA1_11* | 0 | 0.000% |
| *AA_DPB1_8* | 0 | 0.000% |
| *AA_DPB1_9* | 5 | 0.058% |
| *AA_DPB1_11* | 0 | 0.000% |
| *AA_DPB1_33* | 0 | 0.000% |
| *AA_DPB1_35* | 2 | 0.023% |
| *AA_DPB1_36* | 0 | 0.000% |
| *AA_DPB1_55* | 8 | 0.092% |
| *AA_DPB1_56* | 0 | 0.000% |
| *AA_DPB1_57* | 0 | 0.000% |
| *AA_DPB1_65* | 0 | 0.000% |
| *AA_DPB1_69* | 6 | 0.069% |
| *AA_DPB1_72* | 0 | 0.000% |
| *AA_DPB1_76* | 2 | 0.023% |
| *AA_DPB1_84* | 0 | 0.000% |
| *AA_DPB1_85* | 0 | 0.000% |
| *AA_DPB1_86* | 0 | 0.000% |
| *AA_DPB1_87* | 0 | 0.000% |
| *AA_DPB1_91* | 0 | 0.000% |
| *AA_DPB1_96* | 6 | 0.069% |
| *AA_DPB1_170* | 6 | 0.069% |
| *AA_DPB1_178* | 4 | 0.046% |
| *AA_DPB1_194* | 1249 | 14.436% |
| *AA_DPB1_205* | 1248 | 14.424% |
| *AA_DPB1_215* | 1245 | 14.390% |
