# Supplementary material for: MHC-Dependent Mate Selection within 872 Spousal Pairs of European Ancestry from the Health and Retirement Study
Source: Genes (Basel). 2018 Jan 22;9(1):53. doi: 10.3390/genes9010053 (PMC5793204; doi:10.3390/genes9010053)
Supplement: Supplementary file 1 [file genes-09-00053-s001.zip › Table S5.docx]

**Table S5.** Summary of similarity scores (*SC*) for each HLA gene (at 2-digit resolution) in HRS European Americans.

| ***HLA gene*** | ***Similarity Score (SC)^1^*** | ***Similarity Score (SC)^2^*** | | ***Two-sided p-value*** |
| --- | --- | --- | --- | --- |
|  |  | *Mean* | *St dev.* |  |
| *A* | *427* | *438.21* | *14.54* | *0.441* |
| *C* | *414* | *439.67* | *14.55* | *0.078* |
| *B* | *249* | *266.34* | *13.54* | *0.200* |
| *DRB1* | *362* | *358.33* | *15.35* | *0.811* |
| *DQA1* | *693* | *708.29* | *16.18* | *0.345* |
| *DQB1* | *662* | *670.63* | *16.71* | *0.606* |
| *DPA1* | *1306* | *1307.92* | *13.17* | *0.884* |
| *DPB1* | *737* | *733.26* | *13.32* | *0.779* |

^1^Similarity scores between spouses; ^2^Similarity scores summarized from normal distribution.
