# Supplementary figures and images for: MHC-Dependent Mate Selection within 872 Spousal Pairs of European Ancestry from the Health and Retirement Study
Source: Genes (Basel). 2018 Jan 22;9(1):53. doi: 10.3390/genes9010053 (PMC5793204; doi:10.3390/genes9010053)

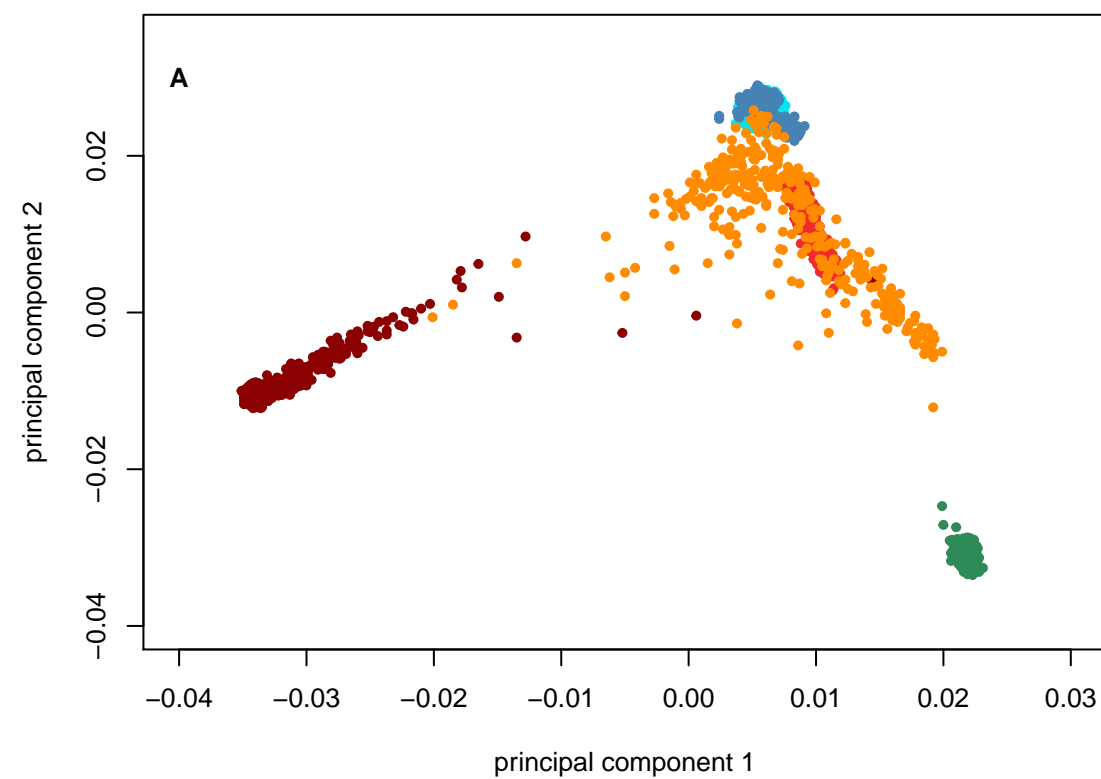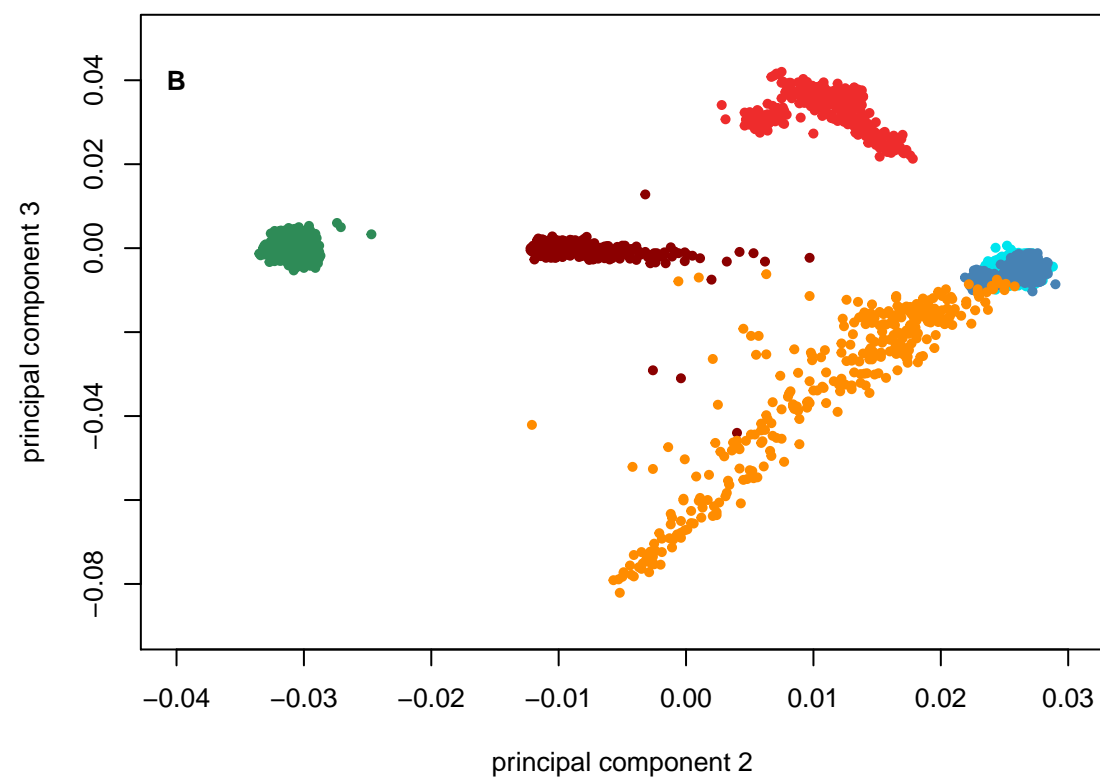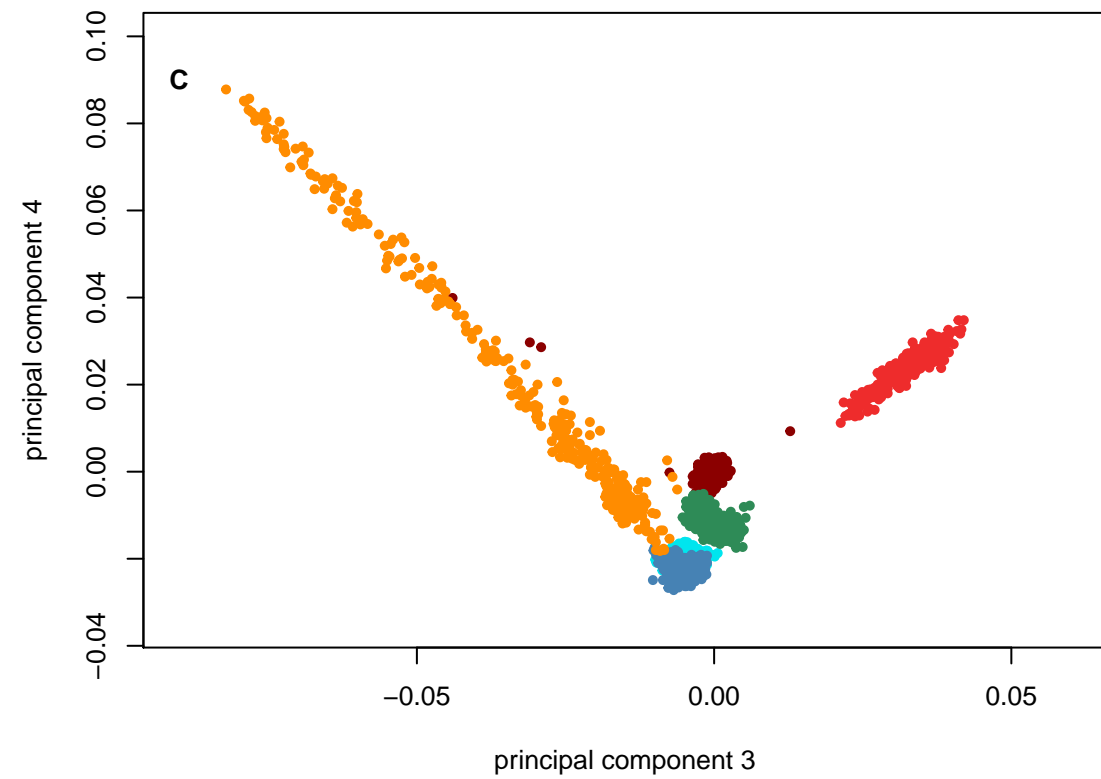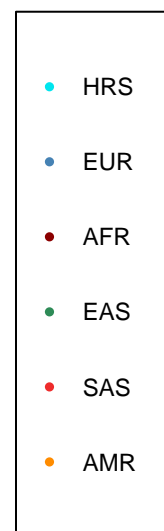

Supplement: Supplementary file 1 [file genes-09-00053-s001.zip › Figure S1.pdf]

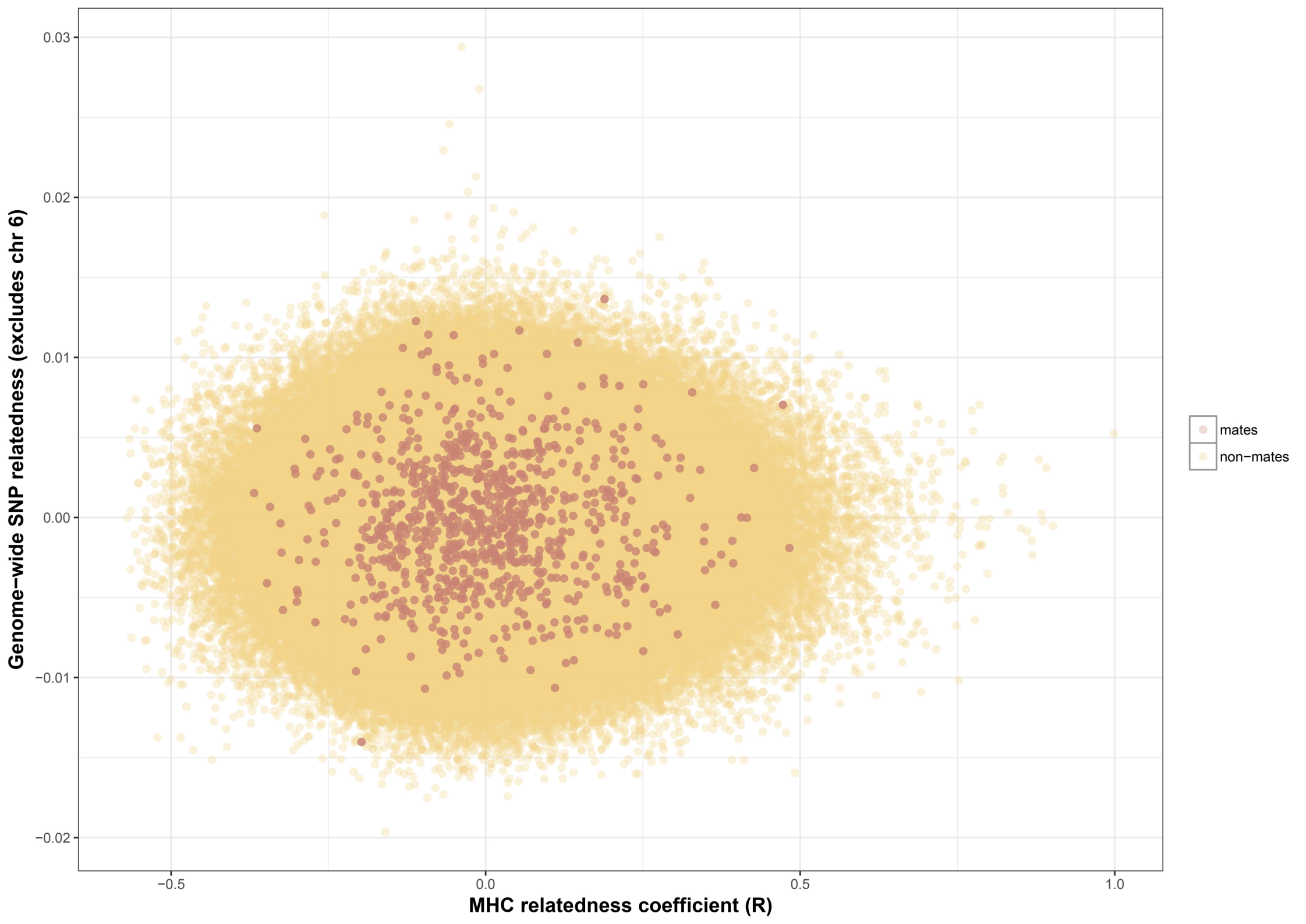

Supplement: Supplementary file 1 [file genes-09-00053-s001.zip › Figure S2.pdf]

**A**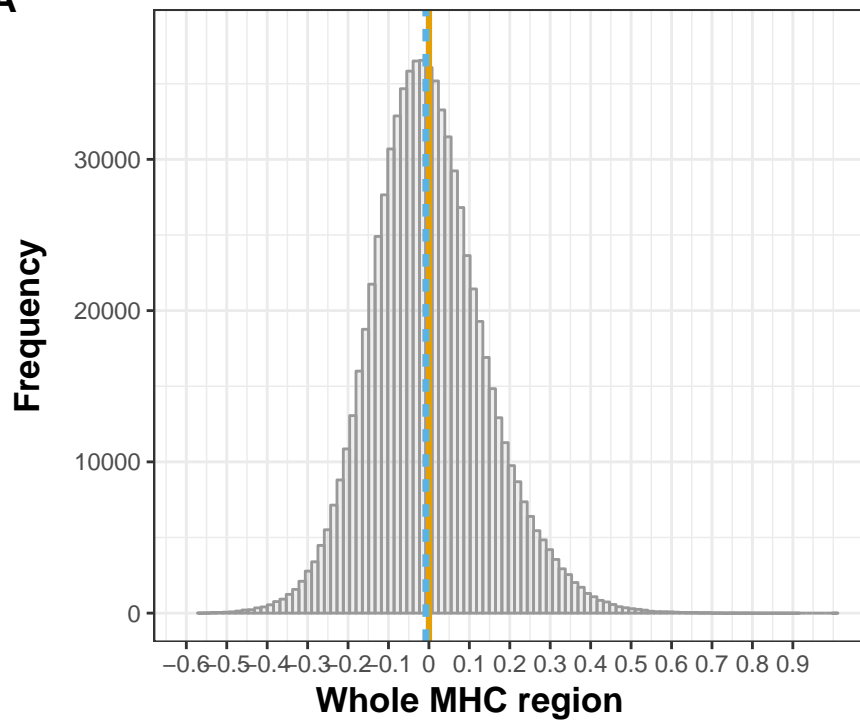**B**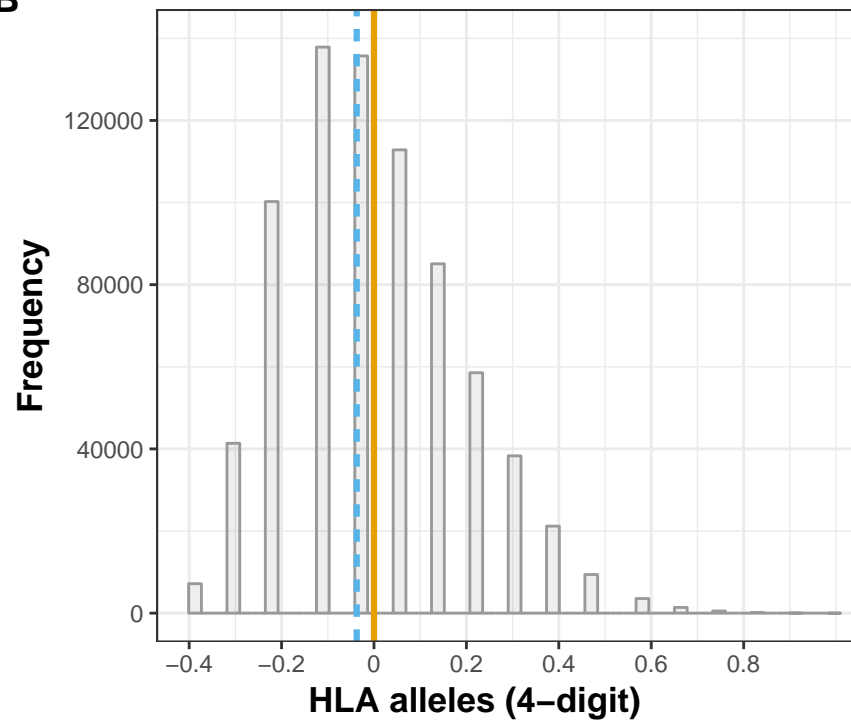**C**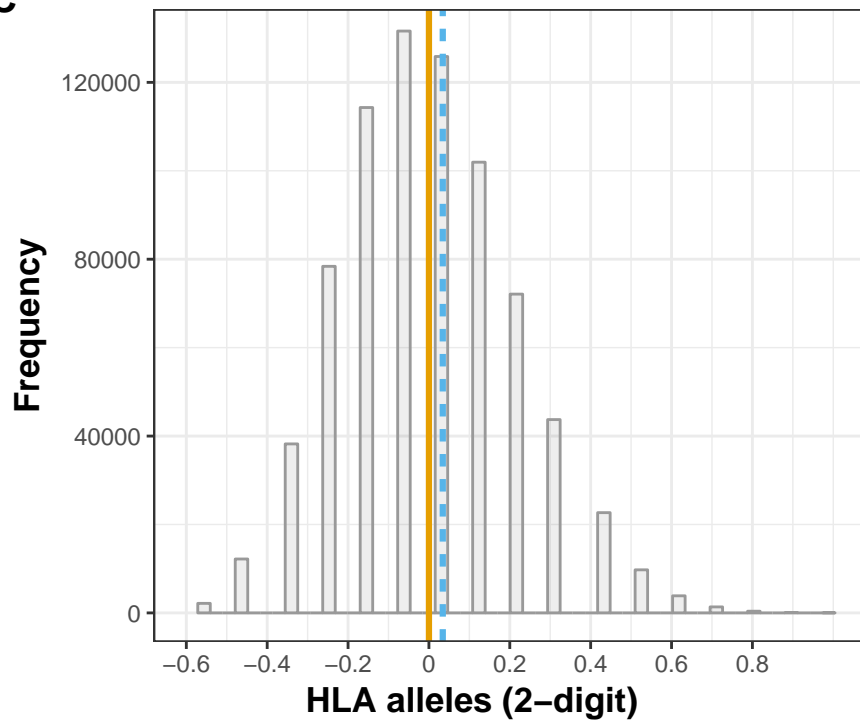**D**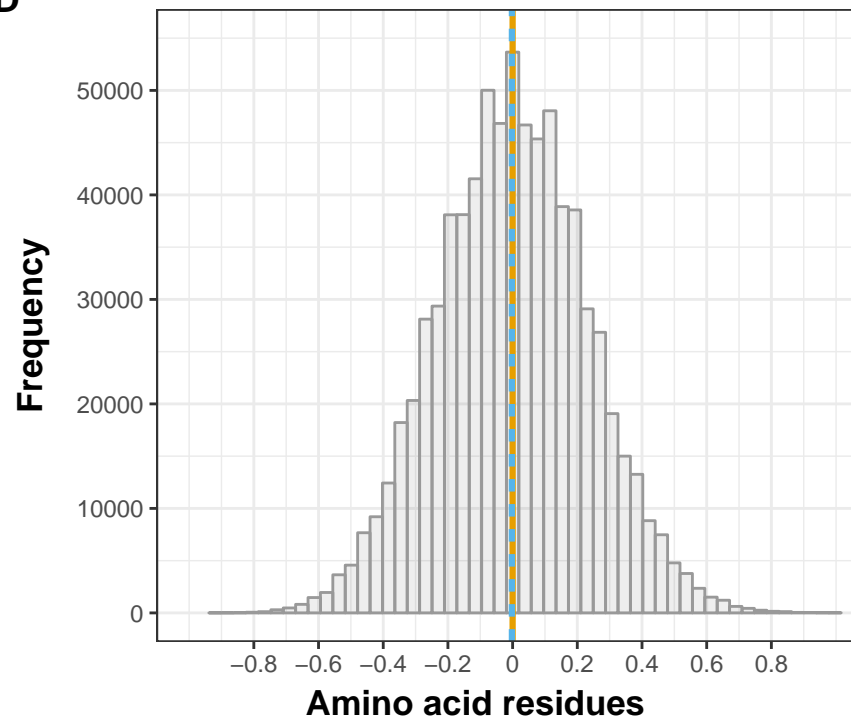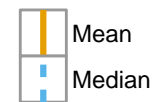

Supplement: Supplementary file 1 [file genes-09-00053-s001.zip › Figure S3.pdf]
